# Identification and Detection of Prokaryotic Symbionts in the Ciliate *Metopus* from Anaerobic Granular Sludge

**DOI:** 10.1264/jsme2.ME15154

**Published:** 2015-12-04

**Authors:** Yuga Hirakata, Mamoru Oshiki, Kyohei Kuroda, Masashi Hatamoto, Kengo Kubota, Takashi Yamaguchi, Hideki Harada, Nobuo Araki

**Affiliations:** 1 Department of Civil Engineering, National Institute of TechnologyNagaoka College, Nagaoka, NiigataJapan; 2 Department of Science of Technology Innovation, Nagaoka University of TechnologyNagaoka, NiigataJapan; 3 Department of Environmental Systems Engineering, Nagaoka University of TechnologyNagaoka, NiigataJapan; 4 Department of Civil and Environmental Engineering, Tohoku UniversitySendai, MiyagiJapan; 5 New Industry Creation Hatchery Center (NICHe), Tohoku UniversitySendai, MiyagiJapan

**Keywords:** *Metopus* ciliates, endosymbionts, *Methanoregula boonei*, *Clostridium aminobutyricum*, anaerobic granular sludge

## Abstract

The aim of the present study was to investigate the prokaryotic community structure of the anaerobic ciliate, *Metopus* sp. using rRNA sequencing, fluorescence *in situ* hybridization (FISH), and transmission electron microscopy (TEM). *Metopus* sp. was physically separated from anaerobic granular sludge in a domestic wastewater treatment plant and anoxically cultivated for 7 d. 16S rRNA gene sequences from the prokaryotes *Methanoregula boonei* and *Clostridium aminobutyricum* were abundantly detected in *Metopus* ciliates. The FISH analysis using the oligonucleotide probes Mg1200b and Cla568 demonstrated that these prokaryotes were localized within *Metopus* cells. These results identify *M. boonei-* and *C. aminobutyricum*-like prokaryotes as novel endosymbionts of *Metopus* ciliates.

Anaerobic ciliates are predators of prokaryotes and influence the abundance, structure, and function (*e.g.*, methanogenesis and sulfate reduction) of prokaryotic communities (4). *Metopus* ciliates are affiliated with the family *Metopidae* of the class *Armophorea*, and are frequently found in anoxic man-made and natural ecosystems including freshwater and marine sediments and landfill sites (6).

Ciliates in anoxic ecosystems harbor endo- and ectosymbiotic prokaryotes. *Metopus* ciliates host endosymbiotic methanogens affiliated with the archaeal genera *Methanobacterium*, *Methanoplanus*, *Methanocorpusculum*, and *Methanosaeta* (14). These endosymbiotic methanogens convert hydrogen or acetate produced by *Metopus* to methane gas (13). The elimination of endosymbiotic methanogens using the specific methanogen inhibitor, 2-bromoethanesulfornic acid, was previously reported to result in a 30% reduction in the growth yield of *Metopus contortus* (7). In addition to endosymbiotic methanogens, ectosymbiotic sulphate-reducing bacteria have been detected in *M. contortus*; however, the molecular phylogeny of these bacteria remains unknown (8). No information is currently available on endosymbiotic bacteria in *Metopus* ciliates. Endosymbiotic bacteria have been detected in anaerobic ciliates, including *Trimyema compressum* and *Cyclidium porcatum*, and have been identified as members of the family *Syntrophomonadaceae* using 16S rRNA gene sequencing. These bacteria may have an as yet uncharacterized physiological role because their removal has been shown to result in 30 to 50% reductions in the growth yield of host ciliates (1, 17). In order to obtain a clearer understanding of symbiosis between prokaryotes and *Metopus* ciliates, the molecular phylogeny of prokaryotic symbionts, particularly endosymbiotic bacteria, needs to be examined in more detail.

Therefore, the aim of the present study was to investigate the molecular phylogeny of endosymbiotic bacteria found in *Metopus* ciliates and demonstrate their symbiosis.

*Metopus* ciliates were anoxically cultivated for 7 d in glucose media. The prokaryotic community structure of *Metopus* cells was subsequently examined in order to screen candidate endosymbionts. *Metopus* ciliates were obtained from anaerobic granular sludge in a domestic wastewater treatment plant (19) using MM-89 and IM-9B micromanipulators (Narishige, Tokyo, Japan), and cultured anoxically at 20°C in ciliate mineral medium containing the following per L of solution: 0.1 g glucose, 0.01 g K_2_HPO_4_, 0.4 g NaHCO_3_, 0.025 g NH_4_Cl, 0.4 g NaCl, 0.2 g MgCl_2_·6H_2_O, 0.15 g KCl, 0.25 g CaCl·2H_2_O, 0.5 g Na_2_S·9H_2_O, 0.5 g L-cysteine hydrochloride monohydrate, 1 mg resazurin sodium salt, 1 mL vitamin solution (16), and 1 mL trace element solution (22). The pH of the media was adjusted to 7.0 with 1N HCl or NaOH. Culture bottles were flushed with nitrogen gas and closed with a butyl rubber stopper. Streptomycin and vancomycin (50 mg L^−1^ each) were also included in the culture medium in order to suppress the growth of free-living and ectosymbiotic bacteria. After 3, 5, and 7 d of cultivation, ten *Metopus* ciliate cells were transferred to 5 μL of sterile distilled water in a sterile PCR tube. After freezing at −80°C and thawing to 60°C three times, the eukaryotic 18S rRNA or prokaryotic 16S rRNA gene sequence was amplified by PCR using the oligonucleotide primers Euk-82F and MedlinB (10, 12) or 515F and 806R (2), respectively. All data were analyzed using QIIME software (version 1.8.0). Sequence reads with low quality scores (Phred quality score <30) were eliminated using the fastx_trimmer tool, and paired-end sequence reads were assembled in the paired-end assembler (llumina, PANDAseq). Nucleic acid sequences with ≥97% similarity were grouped into an operational taxonomic unit (OTU) by the UCLUST algorithm (5). Phylogenetic affiliations of the OTUs were identified using a BLASTN search against reference sequences (NCBI database). In the phylogenetic analysis, partial 18S or 16S rRNA gene sequences were aligned in the ClustalW software and the phylogenetic tree was constructed in MEGA 6.06 software (20) using maximum likelihood (ML; Jones-Taylor-Thornton model), neighbor joining (NJ; Poisson model), maximum parsimony (MP; close neighbor interchange in the random-tree search algorithm), and unweighted pair group methods with the arithmetic mean (UPGMA; a maximal composite likelihood model).

The ciliates cultured in the present study were identified as *Metopus* sp. based on their morphological features as reported previously by Esteban *et al.* (6). The molecular phylogeny of *Metopus* sp. was further examined using PCR-amplified 18S rRNA gene sequences by the Sanger method using a 3730xl DNA Analyzer (Life Technologies). The 18S rRNA gene sequences determined were affiliated with the family *Metopidae*, and sequence similarity between the *Metopus* sp. and *M. contortus* was 97% (Fig. 1a).

The prokaryotic community structure of *Metopus* sp. cells was examined by determining the amplified 16S rRNA gene sequence using the MiSeq sequencer (Illumina, San Diego, CA, USA). A total of 25,683, 30,461, and 29,786 valid prokaryotic sequences were recovered from samples collected after 3, 5, and 7 d of cultivation, respectively (Fig. S1). The most common prokaryotes identified were related to the hydrogenotrophic methanogen *Methanoregula boonei* (16S rRNA gene sequence similarity; 99%) (Fig. 1a) or to the anaerobic bacterium *Clostridium aminobutyricum* (98%) (Fig. 1b), and accounted for 66.1% and 18.7% of total reads at the end of cultivation, respectively. On the other hand, the abundance of the 16S rRNA gene sequence of the sulphate-reducing bacterium, *Desulfovibrio desulfuricans* (16S rRNA gene sequence similarity; 100%) decreased from 7.4% to less than 0.1%. This outcome suggests that *D. desulfuricans* is a free-living or ectosymbiotic bacterium because its growth was compromised by antibiotics.

16S rRNA gene sequences related to *M. boonei* or *C. aminobutyricum* were abundant even after 7 d of cultivation, which suggests that these prokaryotes are endosymbionts of *Metopus* sp.. In order to demonstrate endosymbiosis between *M. boonei-* and *C. aminobutyricum-*like prokaryotes, a fluorescence *in situ* hybridization analysis was performed as described previously (15). *Metopus* sp. cells (Fig. 2a) collected after 7 d of cultivation were fixed with 4% paraformaldehyde for 8 h and hybridized with the oligonucleotide probes Mg1200b (3) and Cla568 (this study) for *M. boonei* and *C. aminobutyricum*, respectively. Samples were then examined using a fluorescence microscope (BX51, Olympus, Tokyo, Japan). The Cla568 probe (5′-ACCTACGCACTCTTTACG-3′) was constructed using the tool Design probes in ARB software (11), and the tool PROBE MATCH from the Ribosomal Database Project was used to examine its coverage and specificity. The optimal formamide concentration of the Cla568 probe was determined to be 20% as described previously (15) using *C. cyclindrosporum* (NBRC13695) and *Stenotrophomonas maltophilia* (JCM3805) cells as a negative control (Fig. S2). When the Mg1200b probe was hybridized, rod-shaped cells (2.3–3.8 μm in length and 0.3–0.4 μm in diameter) were detected in *Metopus* sp. cells (Fig. 2b and c). Rod-shaped cells were abundant in *Metopus* sp. cells (approximately 4,100 cells per ciliate), and oriented parallel to one another. The Cla568 probe detected other rod-shaped cells (2.0–2.5 μm in length and 0.4–0.6 μm in diameter). These cells clustered together and were localized inside *Metopus* sp. cells (100 cells per ciliate) (Fig. 2d, e, and f). We detected Mg1200b- or Cla568-positive rod-shaped cells at various depths within *Metopus* sp. cells, indicating that they were present intracellularly and not simply attaching to the surfaces of *Metopus* sp. cells. The endosymbiosis of prokaryotes in *Metopus* sp. cells was further examined by transmission electron microscopy (Fig. 3a). *Metopus* sp. cells contained a layer of mucocysts beneath the cell membrane, and hydrogenosomes were found next to the mucocyst layer (Fig. 3b) as previously reported in *M. contortus* cells (6). The electron-dense cells of endosymbiotic methanogens were present as neighbors of the hydrogenosomes (Fig. 3b and c). In contrast, endosymbiotic bacterial cells were distributed in the cytoplasm, and some of the endosymbiotic bacteria formed cell clusters (Fig. 3d and e).

A *C. aminobutyricum*-like bacterium was identified as an endosymbiont of *Metopus* ciliates for the first time. Bacteria of the genus *Clostridium* are strict anaerobes and consume sugars and short-chain fatty acids (18, 21). *Metopus* ciliates lack mitochondria, but have a unique organelle called the hydrogenosome in which organic matter is oxidized to hydrogen and volatile fatty acids (*i.e.*, acetate, butyric acid, and lactic acid) (9). It is interesting to speculate that this *C. aminobutyricum*-like bacterium utilizes metabolites supplied from the hydrogenosome; however, additional research is required to confirm this metabolism.

*M. boonei* is a hydrogenotrophic methanogen and may utilize hydrogen produced at the hydrogenosome of *Metopus* sp.. Electron microscopic images (Fig. 3b and c) support this hypothesis; *i.e.*, endosymbiotic methanogens were found next to hydrogenosomes. It is notable that phylogenetically distinct endosymbiotic methanogens have been detected in *Metopus* ciliates; *i.e.*, the relatives of *Methanobacterium*, *Methanoplanus*, *Methanocorpusculum*, *Methanosaeta* (14), and *Methanoregula* (this study) (Fig. 1a). This result indicates that *Metopus* ciliates do not need the phylogenetically specific methanogenic symbionts required by *Trimyema* ciliates (17).

The present study elucidated the prokaryotic community structure of *Metopus* ciliates, and identified *M. boonei-* and *C. aminobutyricum*-like prokaryotes as endosymbionts. Specific interactions between these endosymbionts and the host are important for obtaining a better understanding of the ecophysiology of *Metopus* ciliates because their removal in anaerobic ciliates resulted in 30 to 50% reductions in the growth yield (1, 17). Although the physiological role of endosymbiotic methanogens as hydrogen scavengers has been elucidated previously, future research is required in order to investigate direct and/or indirect interactions between endosymbiotic bacteria and host ciliates.

## Accession numbers

The 18S rRNA gene sequences of *Metopus* sp. and prokaryotic 16S rRNA gene sequences were deposited in the GenBank/EMBL/DDBJ databases under accession numbers LC062508 to LC062510 and LC062148 to LC062381, respectively.

## Supplementary Material



## Figures and Tables

**Fig. 1:**
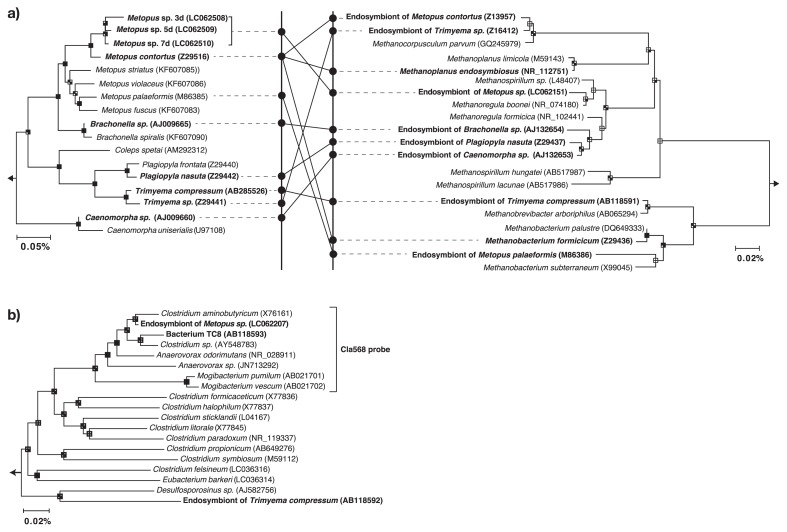
Neighbor-joining tree showing the phylogenetic affiliation of *Metopus* sp., endosymbiotic methanogens (panel a; left and right, respectively), and endosymbiotic bacteria (panel b). Solid lines in the panel represent relationships between endosymbiotic methanogens and host ciliates. Branching points that support a probability of >75% in the bootstrap analyses (based on 1,000 replications, estimated using the NJ method for the upper left sector, the MP method for the upper right sector, the ML method for bottom left sector, and the UPGMA method for the bottom right sector) are shown as black squares. The scale bars represent sequence divergence. The right parenthesis indicates the coverage of the oligonucleotide Cla568 probe designed in the present study.

**Fig. 2:**
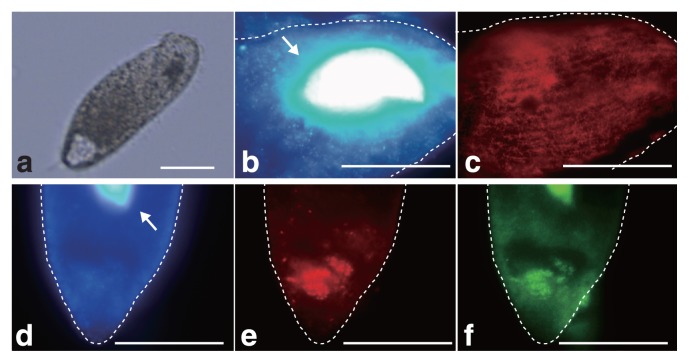
Microscopic observations of *Metopus* sp. (a) and endosymbiotic methanogens (b and c) and bacteria (d–f). Broken lines in b-f indicate the shapes of *Metopus* sp. cells. *Metopus* sp. (a) after 7 d of cultivation was subjected to an *in situ* hybridization analysis. (b and d): DAPI images. The arrow indicates the macronucleus of *Metopus* sp.. (c) Fluorescent micrograph after hybridization with the Mg1200b probe for the endosymbiotic methanogen of *Metopus* sp.. Panels b and c were taken at the same location. (e and f) Fluorescent micrographs after hybridization with the Cla568 or EUB338 probes for endosymbiotic bacteria or most bacteria in *Metopus* sp. cells, respectively. Panels d, e, and f were taken at the same location. The scale bar represents 50 μm.

**Fig. 3:**
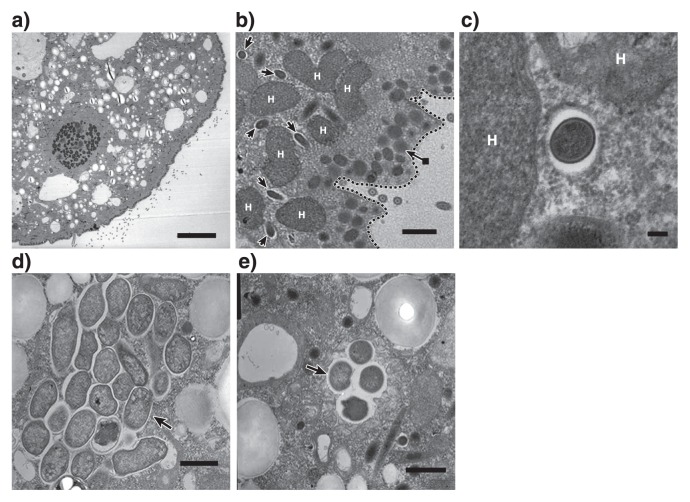
Transmission electron micrographs of *Metopus* sp. cells. (a) A cross-section of *Metopus* sp. cells. Scale bar: 10 μm. (b) Endosymbiotic methanogens (arrows) were detected around a hydrogenosome (H). The broken line indicates the cell membrane, and a layer of mucocysts (an arrow with a closed square symbol at the end) located beneath the membrane. Scale bar: 1 μm. (c) Endosymbiotic methanogens surrounded by a hydrogenosome. Scale bar: 100 nm. (d and e) Endosymbiotic bacterial cells forming bacterial clusters (arrows). Scale bar: 1 μm.
