# Supplementary material for: Identification and Detection of Prokaryotic Symbionts in the Ciliate Metopus from Anaerobic Granular Sludge
Source: Microbes Environ. 2015 Dec 4;30(4):335–8. doi: 10.1264/jsme2.ME15154 (PMC4676557; doi:10.1264/jsme2.ME15154)
Supplement: Supplementary file 1 [file 30_335_s1.pdf]

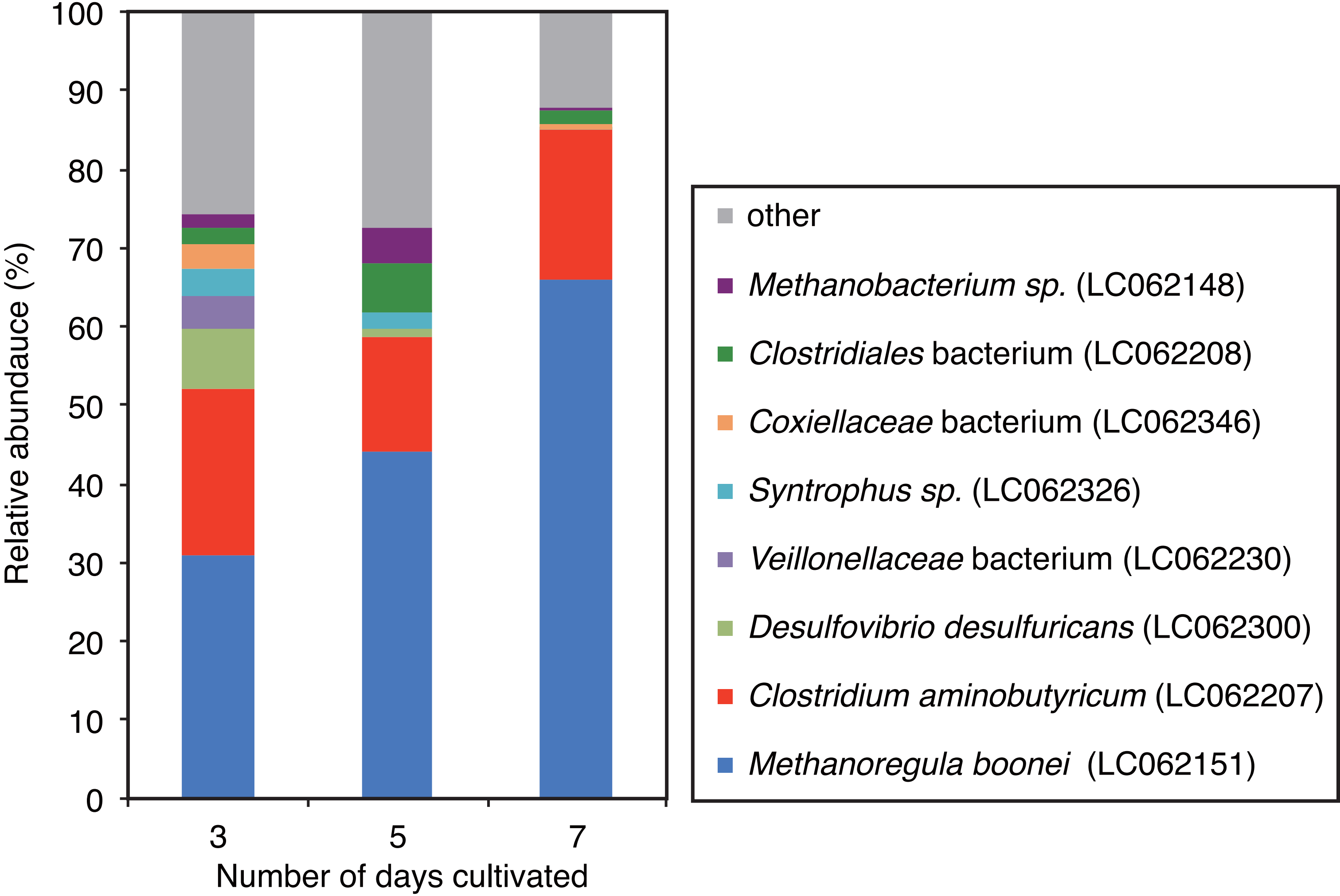

Fig. S1 (Hirakata *et al.*, 2015)

Cla568	ACCTACGCACTCTTTACG
C. cyclindrosporum	ACCTACGCACCCTTTACG
S. maltophilia	ACCTACGCACGCTTTACG

Fig. S2 (Hirakata *et al.*, 2015)
